# Induction of Glucose Metabolism in Stimulated T Lymphocytes Is Regulated by Mitogen-Activated Protein Kinase Signaling

**DOI:** 10.1371/journal.pone.0015425

**Published:** 2010-11-10

**Authors:** Aimee J. Marko, Rebecca A. Miller, Alina Kelman, Kenneth A. Frauwirth

**Affiliations:** Department of Cell Biology and Molecular Genetics and Maryland Pathogen Research Institute, University of Maryland, College Park, Maryland, United States of America; New York University, United States of America

## Abstract

T lymphocytes play a critical role in cell-mediated immune responses. During activation, extracellular and intracellular signals alter T cell metabolism in order to meet the energetic and biosynthetic needs of a proliferating, active cell, but control of these phenomena is not well defined. Previous studies have demonstrated that signaling from the costimulatory receptor CD28 enhances glucose utilization via the phosphatidylinositol-3-kinase (PI3K) pathway. However, since CD28 ligation alone does not induce glucose metabolism in resting T cells, contributions from T cell receptor-initiated signaling pathways must also be important. We therefore investigated the role of mitogen-activated protein kinase (MAPK) signaling in the regulation of mouse T cell glucose metabolism. T cell stimulation strongly induces glucose uptake and glycolysis, both of which are severely impaired by inhibition of extracellular signal-regulated kinase (ERK), whereas p38 inhibition had a much smaller effect. Activation also induced hexokinase activity and expression in T cells, and both were similarly dependent on ERK signaling. Thus, the ERK signaling pathway cooperates with PI3K to induce glucose utilization in activated T cells, with hexokinase serving as a potential point for coordinated regulation.

## Introduction

T cells are dependent on external supplies of glucose to maintain biosynthesis and energy metabolism during activation. Activated T cells adopt a metabolic state of “aerobic glycolysis”, in which glucose flux through glycolysis is high, but only a small proportion of the glucose is oxidized in mitochondria [1]–[5]. A similar phenomenon was recognized in tumor cells more than 80 years ago [6], and was originally thought to represent a defect in mitochondrial function, perhaps as a result of mutations that occurred during oncogenic transformation. However, more recent interpretations suggest that glycolysis is a preferred metabolic pathway for highly proliferative cells, and the shift to a glycolytic phenotype is part of a larger adaptive metabolic program to support growth and proliferation [7]–[9]. Although there is growing appreciation for the importance of metabolic control in both immune responses and tumor development, the pathways that regulate glucose metabolism are still not well defined.

Resting lymphocytes depend upon growth signals from cytokines and low-level T cell receptor (TCR) stimulation in order to maintain metabolic homeostasis [10], [11], whereas CD28 costimulation is required for induction of high level glucose uptake and glycolysis, in large part via activation of the phosphatidylinositol-3-kinase (PI3K)/Akt signaling pathway [12], [13]. The inhibitory receptors cytotoxic T lymphocyte antigen-4 (CTLA-4) and programmed death-1 (PD-1) both block CD28-induced Akt activation, and also prevent the increase in glucose utilization [12], [14], suggesting that regulation of cellular metabolism might be a component of the inhibitory function of these receptors. Strikingly, overexpression of glucose transporter 1 (GLUT1), the major glucose transporter in hematopoietic cells [10], can partially replace costimulation in the induction of proliferation and cytokine production, and constitutively active Akt synergizes with GLUT1 overexpression [13]. Together, these findings indicate the importance of enhanced glucose utilization as a downstream effect of CD28 signaling. However, ligation of CD28 alone does not induce glucose metabolism [12]. Thus, TCR-initiated signaling pathways must cooperate with PI3K/Akt signaling to regulate glucose metabolism.

Ligation of the TCR triggers a variety of signaling cascades, several of which are candidates to regulate metabolism. Three key mitochondrial matrix enzymes, pyruvate dehydrogenase, isocitrate dehydrogenase, and a-ketoglutarate dehydrogenase, are sensitive to calcium levels [15]. This suggests that the rapid influx of calcium that occurs after TCR stimulation may regulate Krebs cycle activity, particularly given the recent evidence that calcium influx in T cells is linked to coordinated mitochondrial calcium uptake [16]. However, since most glucose metabolism in T cells does not utilize the Krebs cycle, it is likely that metabolic regulation by calcium would be more important for alternative Krebs cycle substrates, such as glutamine [17]–[22]. The mitogen-activated protein kinase (MAPK) signaling pathways are also activated by TCR stimulation, and have been implicated in control of glucose metabolism in other cell types, particularly in enhancing glycolysis [23]–[25]. We therefore investigated the role of MAPK signaling in T cell glucose metabolism. We found that the enhanced glucose uptake and glycolysis seen in activated T cells is dependent on extracellular signal-regulated kinase (ERK) signaling, and that this may be due to the regulation of hexokinase expression and activity.

## Results

### Activation of murine T cells leads to enhanced glucose metabolism

**Figure 1:**
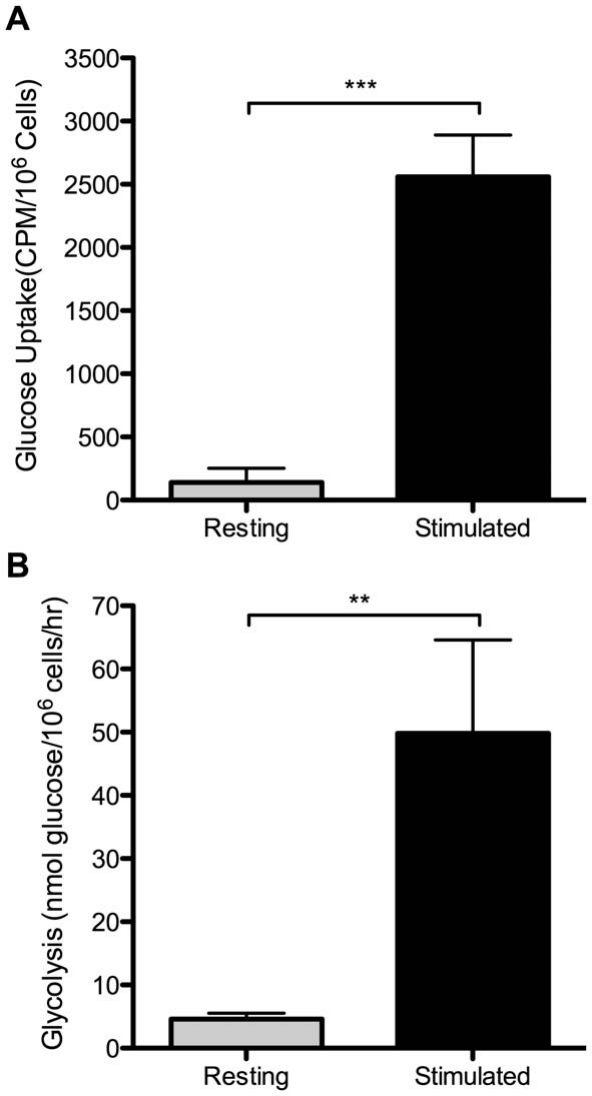
Activation induces an “aerobic glycolysis” phenotype in mouse T cells. Purified T cells were cultured with either control hamster IgG (resting) or anti-CD3 plus anti-CD28 antibodies (stimulated). Glucose uptake (A) and glycolysis (B) rates were measured after 24 hours. ***p<0.0001, means are different; **, p<0.001, means are different. Results are representative of at least 3 independent experiments.

Studies with human peripheral blood T cells have shown that stimulation via mitogenic lectins or CD3/CD28 ligation leads to an "aerobic glycolysis" phenotype, highly inducing glucose uptake and glycolysis [2], [12], [14], [26], [27]. In order to further characterize the regulation of glucose metabolism in T lymphocytes, we decided to switch to the murine system. This would allow us to take advantage of the many genetic and biochemical tools available in the murine system, as well as the lower sample-to-sample variability offered by inbred mouse strains. To confirm that glucose metabolism in murine T cells follows an induction pattern similar to that seen in human T cells, we purified splenic T cells from C57BL/6 mice and stimulated them in vitro with anti-CD3 and anti-CD28 antibodies. After 24 hours of stimulation, glucose uptake by live T cells was measured by the accumulation of radiolabeled 2-deoxyglucose, a non-metabolizable glucose analog, and glycolysis was measured by the generation of ^3^H-labeled H_2_O from ^3^H-labeled glucose, at the step catalyzed by enolase. As shown in Figure 1, activated murine T cells increased both glucose uptake and glycolysis. Thus, primary mouse T cells upregulate glucose utilization upon CD3/CD28 ligation similarly to human T cells, and comparably to previous studies using mitogenic lectins.

**Figure 2 pone-0015425-g002:**
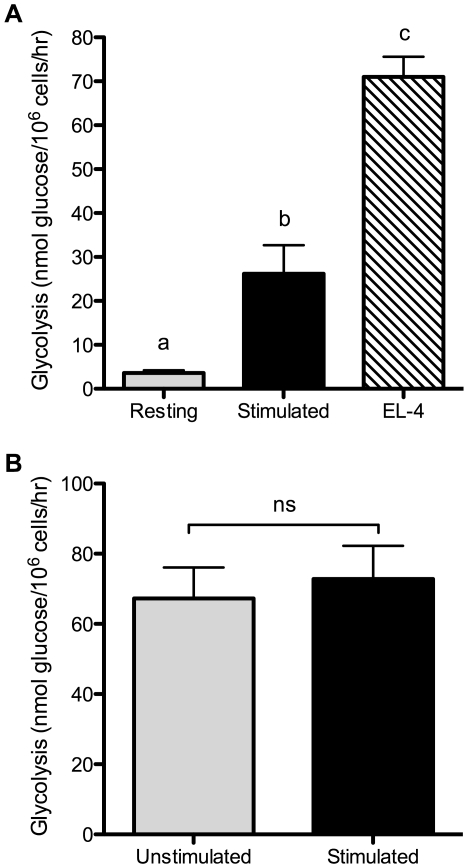
Lymphoma cells show constitutively high glycolysis. (A) Glycolysis was measured in EL-4 lymphoma cells from continuous culture, without additional stimulation. Glycolysis in resting and stimulated T cells (as described in Figure 1) was measured for comparison. p<0.001, means that do not share a letter differ. (B) EL-4 cells were cultured for 24 hours with either control hamster IgG (unstimulated) or anti-CD3 plus anti-CD28 antibodies (stimulated) and glycolysis rates were measured. ns, not significantly different. Results are representative of 2 (A) or 3 (B) independent experiments.

The “aerobic glycolysis” phenotype was originally described in tumor cells [6]. For comparison, we therefore also examined glycolysis in the murine T lymphoma cell line EL-4. EL-4 cells showed a high rate of glycolysis without any TCR stimulation, generally 2–3 times the rate of activated T cells (Figure 2A). This is similar to what we have recently reported for glutamine utilization in EL-4 cells [22]. Thus, in the absence of TCR or CD28 ligation, EL-4 cells display elevated metabolism compared to even fully activated primary T cells. We next tested whether stimulation of EL-4 cells with anti-CD3 and anti-CD28 antibodies would further enhance glycolysis. As shown in Figure 2B, stimulation did not appreciably alter glycolysis in EL-4 cells, indicating that regulation of glucose metabolism has become unlinked from CD3/CD28 signaling.

### Glucose metabolism in activated T cells is regulated by MAPK signaling

Work from several groups has established the importance of the PI3K/Akt pathway in regulating lymphocyte glucose metabolism [12], [13], [28], in addition to its well-known role in insulin receptor-mediated increases in glucose transport [29]–[34]. However, although cross-linking CD28 alone is sufficient to activate PI3K and Akt [35], it is not sufficient to increase glucose metabolism in the absence of TCR/CD3 signaling [12]. Together, these indicate that additional, TCR-induced signaling is required to cooperate with CD28-induced PI3K signals. We therefore examined other signal transduction pathways for regulation of glucose metabolism in activated T cells.

**Figure 3 pone-0015425-g003:**
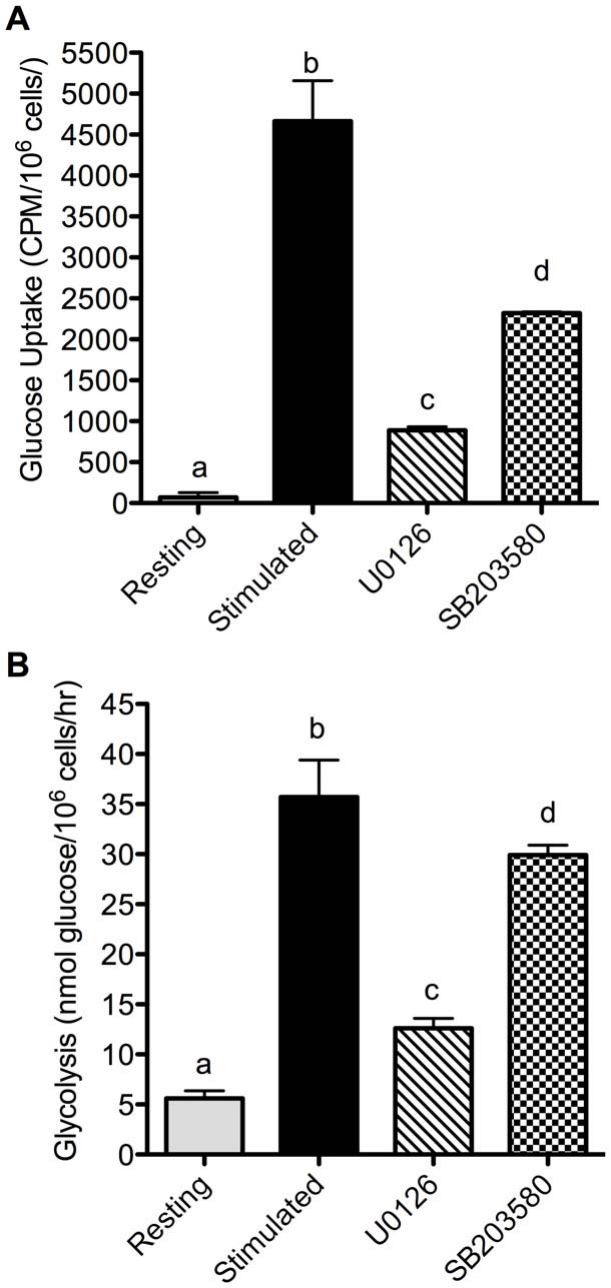
Glucose metabolism in T cells is regulated by MAPK signaling. Purified T cells were stimulated in the absence or presence of the ERK inhibitor U0126 (2.6 µM) or the p38 inhibitor SB203580 (20 µM) for 24 hours. Glucose uptake (A) and glycolysis (B) were measured as in Figure 1. p<0.05, means that do not share a letter differ. Results are representative of 3 independent experiments.

Ligation of the T cell receptor initiates numerous intracellular signaling pathways, including cascades that lead to the activation of MAPK family members. Although well established as regulators of transcription factors during T cell activation, the MAPK pathways have also been implicated in control of amino acid [36]–[38] and glucose [24], [25] utilization in other cell types. We therefore tested the importance of ERK and p38 in the control of glucose metabolism during T cell activation. T cells were stimulated for 24 hours in the presence or absence of specific inhibitors for each of the MAPK pathways, and the effects on glucose uptake and glycolysis were determined. As shown in Figure 3A, the stimulation-induced increase in glucose uptake was almost completely blocked by the ERK inhibitor U0126 (82.2% inhibition), but only partially blocked by the p38 inhibitor SB203580 (51% inhibition). Inhibitors were all used at concentrations that completely blocked T cell proliferation (data not shown) and which do not inhibit other kinases (2.6 µM U0126 [39], and 20 µM SB203580 [40]). As shown in Figure 3B, ERK blockade also largely prevented the induction of glycolysis (76.7% inhibition), whereas p38 inhibition had a much smaller effect on glycolysis (19.3% inhibition). Thus, glucose metabolism appears to be regulated by MAPK signaling, with ERK playing a larger role than p38.

### MAPK signaling controls hexokinase activity

**Figure 4:**
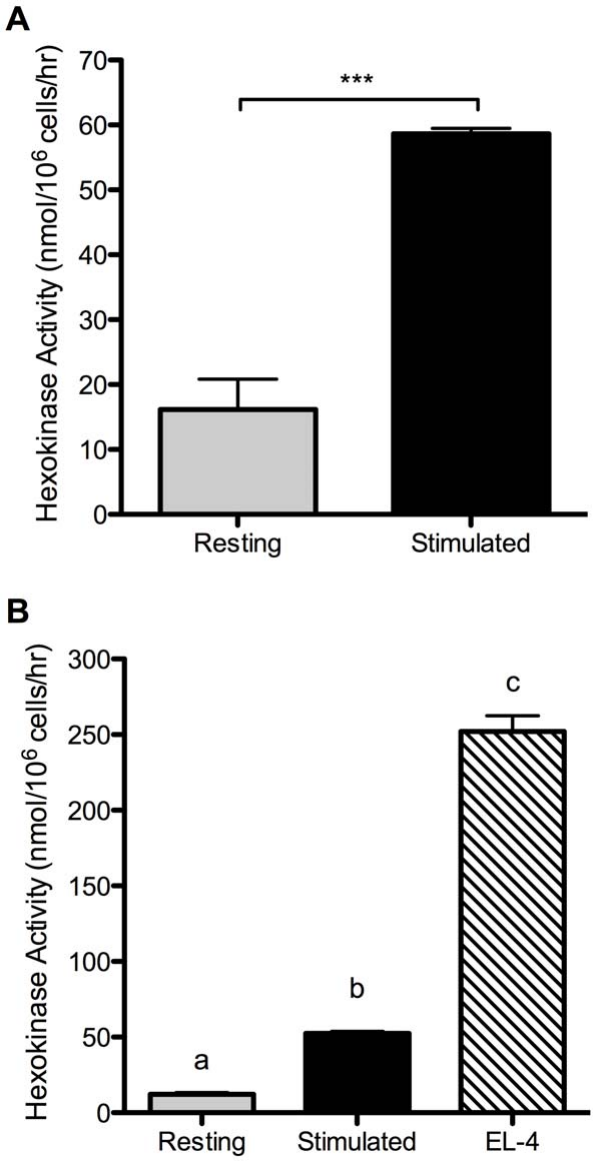
T cell activation induces hexokinase activity. (A) Resting or 24-hour stimulated T cells were lysed in 0.1% Triton X-100 and enzymatic activity of hexokinase was measured. ***p<0.0001, means are different. (B) Continuously growing EL-4 cells were lysed in 0.1% Triton X-100 and hexokinase activity was compared to that in resting and 24-hour stimulated T cells. p<0.0001, means that do not share a letter differ. Results are representative of 3 (A) or 2 (B) independent experiments.

Glucose uptake and subsequent metabolism are dependent on hexokinase function. Phosphorylation of glucose to glucose-6-phosphate traps glucose in the cell, and removes it from equilibrium, allowing continued diffusion through glucose transporters. Glucose-6-phosphate is also the initial substrate for glycolysis. Therefore, hexokinase is well positioned to be a common regulation point. Using an assay for total enzyme activity, we compared hexokinase in resting and activate T cells. As shown in Figure 4A, CD3/CD28 stimulation of T cells strongly increased total cellular hexokinase activity. As with glycolysis, hexokinase activity was high in EL-4 lymphoma cells in the absence of any TCR stimulation (Figure 4B). The hexokinase activity in EL-4 cells was substantially higher than glycolysis rate, which was unlike our findings for primary T cells (compare Figure 1B to [Fig pone-0015425-g002] [Fig pone-0015425-g003] 4A for primary cells, and Figure 2 to [Fig pone-0015425-g003] 4B for EL-4). The disproportionately high hexokinase activity (relative to glycolysis) in EL-4 cells may reflect a large demand in tumor cells for glucose utilization via other metabolic pathways, such as the pentose phosphate shunt [41], although a deeper analysis of glucose metabolism in tumor cells would be required to draw definitive conclusions.

**Figure 5:**
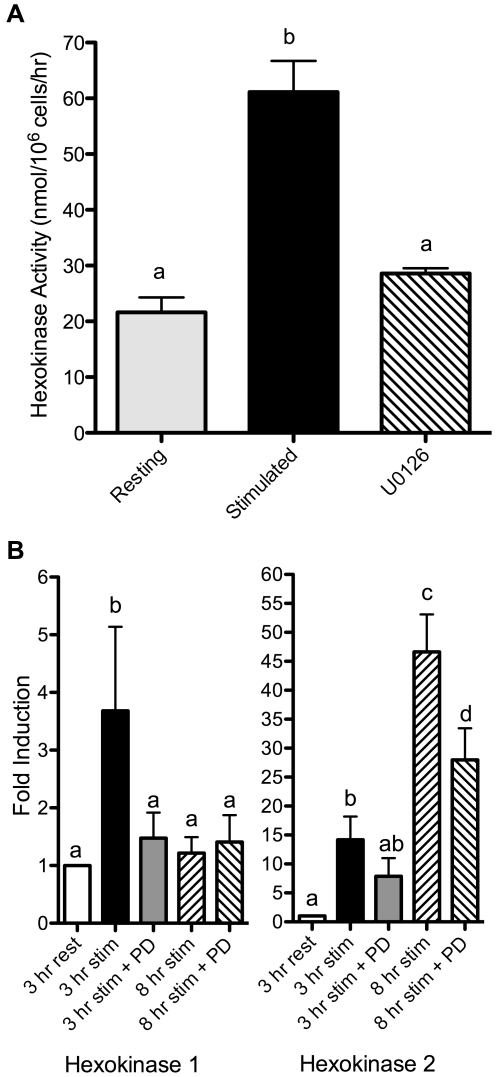
Hexokinase activity in activated T cells is regulated by MAPK signaling. (A) T cells were stimulated in the absence or presence of the ERK inhibitor U0126 (2.6 µM), as in Figure 3, and hexokinase activity was measured. p<0.05, means that do not share a common letter differ. (B) T cells were cultured with control (rest) or anti-CD3 and anti-CD28 antibodies for the indicated times, in the absence (stim) or presence of 40 µM PD98059 (stim + PD). Hexokinase 1 (left panel) and hexokinase 2 (right panel) mRNA levels were determined by quantitative real-time PCR. p<0.05, means that do not share a common letter differ. Results are representative of 3 (hexokinase 1) or 2 (hexokinase 2) independent experiments.

Because hexokinase activity is intimately tied to both glucose uptake and glycolysis, we asked whether induction of hexokinase also required MAPK signaling. We focused on ERK, as inhibition of this pathway had much greater effects than p38 inhibition on uptake and glycolysis. In addition, ERK has been reported to positively regulate hexokinase in cultured cells [23]. Using the ERK inhibitor U0126, we found that induction of hexokinase activity downstream of TCR/CD28 ligation was strongly dependent on ERK signaling (Figure 5), similar to glucose uptake and glycolysis. Comparable results were seen using a second ERK inhibitor, PD98059 (data not shown). Thus, by regulating hexokinase activity, the ERK signaling pathway is able to control glucose metabolism in a coordinated fashion.

We recently reported that ERK positively regulates mRNA expression of the enzyme glutaminase, which catalyzes the first step in glutamine metabolism [22]. We therefore tested whether ERK signaling in T cells also induces hexokinase gene expression, analyzing the same mRNA samples that we used previously to study glutaminase expression. T cell stimulation increased mRNA levels of hexokinase 1 and 2 within 3 hours, and hexokinase 2 consistently showed higher and longer-lived induction than hexokinase 1 (Figure 5B). Stimulation of T cells in the presence of 40 µM PD98059, another highly specific ERK inhibitor [42], ablated the increase in hexokinase 1 mRNA expression, and partially blocked the increase in hexokinase 2 mRNA (Figure 5B). Thus, glucose metabolism in T cells appears to be regulated, at least partly, by ERK control of hexokinase gene expression.

## Discussion

T cell activation is accompanied by a large increase in glucose uptake and glycolysis, which is necessary to support new metabolic demands. Previous studies have shown that the enhanced glucose utilization is dependent on signals from the costimulatory receptor CD28, and particularly the PI3K/Akt pathway [12], [14]. However, CD28 ligation alone does not induce changes in glucose metabolism, despite strongly activating PI3K and Akt [12], [35], implicating TCR-initiated signaling in this process as well. In this study, we found that the MAPK family member ERK plays an important role in the regulation of glucose metabolism during T cell activation, while p38 makes a smaller (and perhaps redundant) contribution. Blockade of ERK signaling strongly inhibited increases in glucose uptake and glycolysis. High level glucose metabolism has been shown to be required for the survival, proliferation, and cytokine production (especially IFN-γ) of activated T cells [13], [43]. Thus, in addition to regulating gene expression via the activation of transcription factors, ERK also controls T cell function by enhancing glucose metabolism.

Glucose metabolism is controlled by a combination of allosteric activators and inhibitors, as well as by post-translational modification of key enzymes. This provides a variety of potential targets for MAPK regulation. Hexokinase, which catalyzes the phosphorylation of glucose to glucose-6-phosphate, is a highly attractive candidate, for several reasons. Glucose uptake in lymphocytes occurs by facilitated diffusion through GLUT1, meaning that glucose can only move down a concentration gradient. Upon phosphorylation by hexokinase, glucose is removed from equilibrium and simultaneously trapped inside the cell. Thus, glucose uptake is critically dependent on hexokinase activity. In addition, phosphorylation is a required step for both glucose storage as glycogen, and glucose catabolism via glycolysis and the pentose phosphate pathway. Thus, hexokinase is likely to be a gatekeeper for cellular glucose consumption. We found that resting T cells have low hexokinase activity, which is highly induced upon activation. Strikingly, hexokinase activity levels corresponded closely to glycolysis rates, reinforcing the idea that hexokinase represents a limiting factor for glucose metabolism in T cells. As with glucose uptake and glycolysis, induction of hexokinase activity was dependent on ERK signaling, providing a point for coordinated control of glucose metabolism.

The finding that hexokinase activity in T cells is regulated by MAPK signaling also provides insight into the cooperation between the TCR and CD28 in enhancing glucose utilization. Studies of the mechanism of growth factor receptor inhibition of cell death revealed that hexokinase is a target of Akt, with evidence that Akt can both induce subcellular redistribution and increase enzymatic activity of hexokinase [44], [45]. Thus, the CD28 signal (Akt) and TCR signals (ERK) intersect at hexokinase. It is unknown whether hexokinase is a direct phosphorylation target for Akt or ERK, but regulation of hexokinase activity by ERK in glomerular mesangial cells appears to require de novo gene transcription [23], consistent with our own finding that induction of hexokinase gene expression during T cell activation is inhibited by blocking ERK signaling. However, we also found that expression of hexokinase 2 was only partially dependent on ERK, suggesting that the mechanism may involve changes in both hexokinase expression level and post-translational modifications. Further investigation will be necessary to sort out these possibilities.

A model of aerobic glycolysis, with glutamine serving as a critical Krebs cycle substrate, has been proposed to be a common feature of most or all rapidly growing and dividing cells, including tumor cells [7]–[9]. Indeed, we found that the EL-4 T lymphoma cell line demonstrates a pattern of glucose metabolism that is similar to that of activated primary T cells, but with the various aspects of glucose utilization further elevated. This is also similar to what we recently reported for glutamine metabolism in EL-4 cells vs. activated normal T cells [22]. Thus, the metabolic alterations that have long been observed in tumors (and which are now used diagnostically to detect cancer) may not be due to metabolic dysfunction, but rather may represent an adaptation in which increases in cellular metabolism occur without the normal requirement for receptor-mediated induction.

## Materials and Methods

### Ethics Statement

Animals used in this study received humane care in strict compliance with the “Guide for the Care and Use of Laboratory Animals” of the National Institutes of Health. All protocols were approved by the Institutional Animal Care and Use Committee of the University of Maryland (Permit Number R-09-70).

### Antibodies and reagents

Anti-CD3 (mAb 145-2C11) and anti-CD28 (mAb 37.51) antibodies, control hamster IgG, and phycoerythrin-labeled anti-Thy1.2 antibodies were purchased from eBioscience (San Diego, CA). U0126, PD98059, and SB203580 were purchased from Biomol (Plymouth Meeting, PA). Hexokinase (Enzyme Commission 2.7.1.1) was from MP Biomedicals (Santa Ana, CA). Glucose-6-phosphate dehydrogenase (Enzyme Commission 1.1.1.49), ATP, and NADPH were from Sigma-Aldrich (St. Louis, MO). 3-mercapto-1,2-propanediol (thioglycerol) was purchased from Fisher Scientific (Fairlawn, NJ). [^3^H]-2-deoxyglucose, 5-[^3^H]-glucose, and [^3^H]-H_2_O were from PerkinElmer (Shelton, CT).

### Animals

C57BL/6J mice (6 weeks old) were purchased from The Jackson Laboratory (Bar Harbor, ME). All mice were maintained in ventilated microisolator cages (Animal Care Systems, Centennial, CO) in the University of Maryland animal facility. Mice were euthanized by carbon dioxide inhalation, as recommended by the American Veterinary Medical Association Panel on Euthanasia, and all efforts were made to minimize suffering.

### T cell purification

Murine T cells were purified from spleens using the EasySep Mouse T Cell Enrichment Kit (Stem Cell Technologies, Vancouver, BC, Canada) or the Dynal Mouse T Cell Negative Isolation Kit (Invitrogen, Carlsbad, CA) according to the manufacturer's protocol. Purified T cells were generally >95% Thy1-positive, as determined by flow cytometry.

### Cell lines and culture

The murine EL-4 thymoma cell line was purchased from American Type Tissue Collection (Manassas, VA). All cells were maintained in RPMI1640 medium (Mediatech, Manassas, VA) supplemented with 10% FBS (Hyclone, Logan, UT), penicillin/streptomycin, 10 mM HEPES buffer, 2 mM glutamine, and 55 µM 2-mercaptoethanol at 37°C in a 5% CO_2_ atmosphere.

### T cell stimulation

Anti-CD3 and anti-CD28 antibodies were covalently attached to tosyl-activated Dynabeads (Invitrogen) following the manufacturer's instructions. Beads were mixed with T cells at a 3∶1 ratio of beads:cells and incubated at 37°C for the desired time. For unstimulated samples, T cells were mixed with control hamster IgG-linked Dynabeads, and were then cultured in parallel with stimulated samples. For experiments with MAPK inhibitors, the inhibitors were added to culture medium at the time of stimulation, and were present throughout the incubation.

### Glucose uptake, glycolysis, and hexokinase assays

Glucose uptake and glycolysis were measured as described previously [12]. To measure hexokinase activity, purified T cells were lysed in 0.1% Triton X-100 at a concentration of 2×10^8^ cells/mL, and 5 µL of lysate was used for each reaction. The assay was carried out as described by Rathmell et al.[45], in a final volume of 100 µL. Activity was determined by the change in absorbance at 340 nm, using a VersaMax spectrophotometer and SoftMax Pro software (Molecular Devices). OD readings were taken every 6–10 s for at least 30 minutes, and rate was calculated from the linear portion of the curve. A_340_ values were converted into substrate concentrations using Beer's Law (A = εLc, where A is absorption, ε is the extinction coefficient, L is the path length, and c is concentration) and the extinction coefficient of NAD(P)H (6.22 mM^−1^ cm^−1^). Data points for all analyses are presented as the mean of triplicate samples ± S.D.

### Real-time PCR

Total RNA was extracted from unstimulated or stimulated T cells using the Nucleospin RNA II kit (Macherey-Nagel, Düren, Germany) or the RNeasy Mini kit (Qiagen, Valencia, CA). cDNA was generated using iScript reverse transcriptase reagents (Bio-Rad, Hercules, CA). Primers for real-time PCR were designed using the Primer-BLAST tool of the National Center for Biotechnology Information (http://www.ncbi.nlm.nih.gov/tools/primer-blast/), and were as follows: hexokinase 1, forward 5′ GCCACGCTCGGTGCCATCTT 3′, reverse 5′ GGTCTTGTGGAACCGCCGGG 3′; hexokinase 2, forward 5′ AGCTCTGTGGCGCAGGCATG 3′, reverse 5′ TCGGACAGGCCACAGCAGTG 3′. Real-time PCR was performed in an iCycler iQ system (Bio-Rad) using the SensiMix SYBR & Fluorescein kit (Bioline, Taunton, MA) and analyzed with MyiQ software (Bio-Rad). Melt curve analysis was performed to confirm the presence of a single PCR product for each reaction, and agarose gel electrophoresis was used to confirm that PCR products were the expected sizes. Fold induction was calculated by the ΔΔCt method, using 18S rRNA as the reference. Data are presented as the means of triplicate samples ± SD.

### Statistics

All statistical analyses were performed using Prism Version 5 software (GraphPad, San Diego, CA). The minimal level of confidence at which experimental results were considered significant was p<0.05. Statistical significance between samples was determined by one-way analysis of variance (ANOVA) with Bonferroni post-test analysis, or by two-tailed t test.
